# CX-5461 Enhances the Efficacy of APR-246 via Induction of DNA Damage and Replication Stress in Triple-Negative Breast Cancer

**DOI:** 10.3390/ijms22115782

**Published:** 2021-05-28

**Authors:** Ashwini Makhale, Devathri Nanayakkara, Prahlad Raninga, Kum Kum Khanna, Murugan Kalimutho

**Affiliations:** QIMR Berghofer Medical Research Institute, 300 Herston Road, Herston, Brisbane, QLD 4006, Australia; Ashwini.Makhale@petermac.org (A.M.); Devathri.Nanayakkara@qimrberghofer.edu.au (D.N.); Prahlad.Raninga@qimrberghofer.edu.au (P.R.)

**Keywords:** triple-negative breast cancer, p53, combination therapy, APR-246, CX-5461, DNA damage response, apoptosis, cell cycle

## Abstract

Triple-negative breast cancer (TNBC) is an aggressive subtype of breast cancer lacking targeted therapy. Here, we evaluated the anti-cancer activity of APR-246, a P53 activator, and CX-5461, a RNA polymerase I inhibitor, in the treatment of TNBC cells. We tested the efficacy of individual and combination therapy of CX-5461 and APR-246 in vitro, using a panel of breast cancer cell lines. Using publicly available breast cancer datasets, we found that components of RNA Pol I are predominately upregulated in basal-like breast cancer, compared to other subtypes, and this upregulation is associated with poor overall and relapse-free survival. Notably, we found that the treatment of breast cancer cells lines with CX-5461 significantly hampered cell proliferation and synergistically enhanced the efficacy of APR-246. The combination treatment significantly induced apoptosis that is associated with cleaved PARP and Caspase 3 along with Annexin V positivity. Likewise, we also found that combination treatment significantly induced DNA damage and replication stress in these cells. Our data provide a novel combination strategy by utilizing APR-246 in combination CX-5461 in killing TNBC cells that can be further developed into more effective therapy in TNBC therapeutic armamentarium.

## 1. Introduction

Breast cancer (BC) is the second most common malignancy exhibiting disparate clinical, pathological and molecular attributes with diverse response to treatment [1,2,3]. Triple-negative breast cancers (TNBCs) are a BC subtype characterized by the lack of hormonal receptors (estrogen and progesterone) and HER2 amplification. Out of all the diagnosed cases worldwide, TNBCs account for 10–20% of the cases, with 75% classified under ‘basal-like’ [1,2,3]. TNBCs often occur in premenopausal women under 40 years old, and show ethnic predisposition toward women of African-American or Hispanic origin [4]. Being biologically aggressive, TNBCs often metastasize to the lungs and brain. TNBC patients exhibit a good initial response to neoadjuvant chemotherapy, but the disease-free survival period is significantly shorter with a high relapse rate, with only 30–45% of patients attaining a complete pathological response (pCR) to chemotherapy [1,5]. Currently, there is a dearth of targeted therapies in the treatment of TNBCs and, therefore, multiple efforts are in progress in unearthing effective therapeutic strategies, either as a single agent or as combination therapy [1,6,7,8].

*TP53* is major tumor suppressor gene that governs diverse cellular responses, including DNA damage, hypoxia, nutrient starvation and oncogenic suppression through transcriptional control of target genes [9,10,11]. However, *TP53* is the most frequently mutated gene in cancers, particularly in TNBCs [12], which accounts for 80–90% cases. Mutation in *TP53* confers a high rate of genetic instability and reduced overall survival (OS) [13]. A large fraction of mutations occurring in the *TP53* gene are missense mutations clustered in the DNA-binding domain, which disrupt sequence-specific DNA binding and results in loss of wild-type p53 activity [14,15]. Mutant *TP53* can also have gain-of-function (GOF) activity to induce tumorigenesis [16,17,18]. In particular, tumor cells with mutant p53 circumvent senescence and apoptosis, which is associated with increased resistance to conventional chemotherapy. Over the years, several drugs have been developed to target mutant-p53. Of these, PRIMA-1^MET^ (p53-dependent reactivation of massive apoptosis) (also known as APR-246), is a pro-drug that generates active compound methylene quinuclidinone (MQ), which binds cysteine residues in the DNA-binding domain of mutant-p53, resulting in refolding into wild-type-p53 confirmation and subsequent restoration of tumor suppressor activity in a number of cancer models [19,20]. APR-246 induces cell death either by (a) binding directly to mutant p53 to promote proper protein folding to restore normal function, or (b) dissociation of the interaction between p53 and its family members (p63 and p73) [21]. More recent studies provide evidence for the p53-independent anti-tumor effects of APR-246 through inhibition of glutathione synthesis, and thioredoxin reductase that leads to altered redox balance to promote ROS production prior to induction of cell death [22,23,24]. Importantly, APR-246-induced cell death can be rescued by treating tumor lines with antioxidants, demonstrating the crucial role of ROS in APR-induced cell death. APR-246 is currently undergoing a Phase IIb clinical trial in high-grade serous ovarian cancer (HGSOC) in combination with doxorubicin (ClinicalTrials.gov Identifier: NCT03268382).

Increased transcription of ribosomal RNA genes by RNA polymerase I (POL I) has been shown to be a common feature in human cancers [25,26]; hence, targeting this pathway is very specific to cancer cells. CX-5461 was initially developed as a highly potent small-molecule inhibitor of rRNA synthesis in tumor cells [27]. It selectively and specifically inhibits the RNA Pol I-driven transcription of rRNA in cancer cells but does not inhibit RNA Pol II-driven mRNA transcription, DNA replication or protein synthesis [28,29]. In cancer cells, CX-5461 induces death by various mechanisms [26,28]. Currently, CX-5461 is in Phase I/II clinical trials in hematologic (Trial ID: ACTRN12613001061729) and triple negative and BRCA-deficient breast cancer (Canadian Cancer Trials Group ID: NCT02719997). CX-5461 has been recently demonstrated to be effective in killing BRCA 1/2-deficient cells by inducing stabilization of the G4 quadraplex structures where resolution requires a BRCA 1/2-dependent DNA damage repair pathway [30].

The cytotoxic effects of APR-246 and CX-5461 as single agents in clinical trials prompted us to study the effect of these drugs in TNBC cells. Given that APR-246 can reactivate mutant p53 to wild-type protein conformation, and CX-5461, through nucleolar disruption, prevents MDM2 from binding to the p53 protein in the nucleolus, we hypothesized that combination therapy would prevent wild-type p53 protein from being degraded by MDM2, resulting in apoptosis. Here, we demonstrate that components of Pol I transcription are selectively enriched in basal-like breast cancer, which renders them sensitive toward Pol I inhibition in TNBC cell lines compared to other subtypes of breast cancer. Combination treatment with APR-246 and CX-5461 causes an increased in DNA damage and replication stress, leading to apoptosis, which could be a novel therapeutic strategy in targeting TNBC.

## 2. Results

### 2.1. RNA Polymerase I Transcriptional Alteration Predicts Poor Clinical Outcome in Breast Cancer

**Figure 1:**
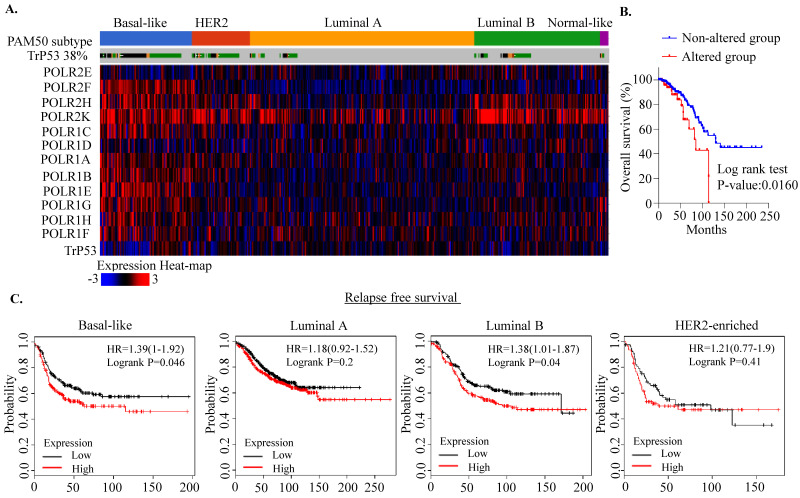
RNA polymerase I transcription alterations in breast cancer. (**A**) Heatmap analysis of components of RNA Pol I transcription at the mRNA level in TCGA breast cancer samples. Patient samples were subdivided into PAM50 subtypes. Data were derived from cbioportal (http://www.cbioportal.org/ (accessed on 1 March 2021)). (**B**) Breast cancer TCGA patient’ data were divided into two subgroups based on their expression patterns (whether altered or non-altered) and survival probability was plotted. (**C**) Kaplan–Meier survival analysis between components of RNA Pol I transcription alteration and clinical outcomes in breast cancer patients, using the KMplotter dataset (http://kmplot.com/ (accessed on 1 March 2021)). The expression pattern was stratified on relapse-free survival.

The RNA Pol I-dependent pathway is significantly altered in cancers [26]. Pol I is a multi-subunit complex comprising 14 polypeptide subunits of which only 13 have been identified so far in humans [31]. However, very little is known concerning alteration of components of this complex in breast cancer pathogenesis in a p53 mutant context. Thus, we first explored the breast cancer subtype-specific expression patterns of RNA Pol I transcription, using The Cancer Genome Atlas (TCGA) and Molecular Taxonomy of Breast Cancer International Consortium (METABRIC) datasets. We found that components of RNA Pol I are markedly altered at the transcript levels in TCGA basal-like breast cancer compared to non-basal-like breast cancer without significant copy number alterations (Figure 1A and Figure S1A). As expected, these alterations are mainly observed in mutant P53 tumors, compared to non-mutant tumors (Figure 1A). Moreover, among these components, POLR2K seems to be markedly altered amongst all breast cancer subtypes, with 11% of cases exhibiting amplification (Figure 1A and Figure S1A). Notably, patients with Pol I component alterations exhibited poor clinical outcomes compared to patients without alterations (log rank test *p*-value: 0.0160; Figure 1B). Likewise, a similar trend in changes in Pol I transcript levels were also observed using the METABRIC dataset, albeit with a higher amplification rate of POLR2K copy number (22%) (Figure S1B). Consistently, we observed that patients with Pol I component alterations exhibited poor overall and relapse-free survival, similar to TCGA patients (Figure S1C). Moreover, analysis using METABRIC 10–IntClust-based classification identified that IntClust 1, 9 (luminal poor prognosis), IntClust 5 (HER2+) and IntClust 10 (basal-like) groups have more pronounced Pol I alterations compared to other subgroups (Figure S1D). Likewise, the basal and luminal B subgroups with alterations of components of RNA Pol I exhibited poor relapse-free survival (Figure 1C). In order to further identify the role of Pol I in breast cancer, we analyzed the shRNA datasets that exhibit an essentiality score (GARP score) required for cell viability in a large panel of cancer cell lines [32]. We found that knocking down some of the Pol I components, highlighted in red boxes, exhibited a trend toward a lower GARP essentiality scores in TNBC cell lines compared to other subtypes (Figure S2). Collectively, these data suggest that RNA Pol I-driven pathway is significantly altered in the TNBC and TP53 context; knocking down some of the component seems to be essential for the survival of TNBC cells, thus could be a viable option in targeting this pathway in breast cancer. 

### 2.2. APR-246 and CX-5461 Combination Enhances Growth Suppression Effect in a Panel of Breast Cancer Cell Lines

**Figure 2:**
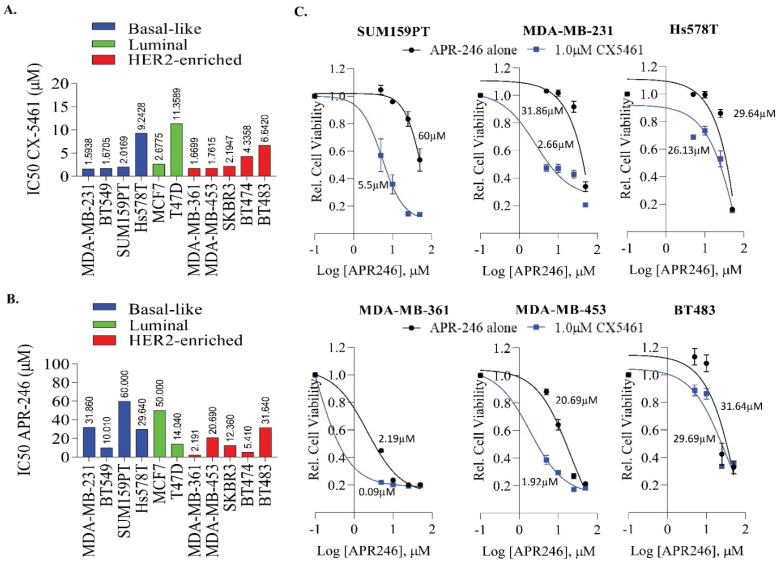
Impact of CX5461 and APR-246 treatment on cell growth in a panel of breast cancer cell lines. (**A**) A panel of breast cancer cell lines were used to determine the IC50 value of CX-5461. Cells were exposed to different concentrations of CX-5461 and cell viability was determined after 6 days, using MTS assays. The dose-response curve was generated by calculating cell viability relative to untreated control and plotted against drug concentration, *n* =  2–3 experiments and data were pooled together to generate IC50. (**B**) The IC50 of APR-246 was determined in a panel of breast cancer cell lines. (**C**) Representative cell lines as indicated in the figure were exposed to different concentrations of APR-246 (5–25 μM) alone or in combination with 1 μM of CX-5461 and cell viability was determined as described in A.

In order to further evaluate the effect of RNA Pol I inhibition in breast cancer, we used a well-known small-molecule inhibitor, CX-5461 and assessed cell viability across a number of breast cancer cells lines over a period of six days. We found that most of the breast cancer cell lines were sensitive to CX-5461 irrespective of subtype (Figure 2A), except Hs578T (9.24 µM), T47D (11.35 µM), BT474 (4.33 µM) and BT483 (6.64 µM). The IC50 ranged from ~1.5 µM to 11.35 µM. These data suggest that CX-5461 has off-target effects in addition to targeting the components of the RNA Pol I transcription machinery which are essential in TNBCs. Consistent with this notion, CX5461 has recently been shown to act as a topoisomerase II poisoning drug [33] and G4 quadruplex stabilizing agent [30]. In addition, we also assessed the effect of APR-246 as a single agent in this panel (Figure 2B). We found that most of the TNBC cell lines which harbor p53 mutation exhibit moderate sensitivity to APR-246, ranging from 10 µM to 52.35 µM. However, HER2+ breast cancer cell lines exhibited much better sensitivity toward APR-246 (ranged from 3.9 µM to 22.20 µM) (Figure 2B). We then assessed the combinatorial effect of APR-246 with CX-5461 in selective breast cancer cell lines both with and without *TP53* (Figure 2C and Figure S3). We found that addition of 1 µM of CX-5461 enhances the efficacy of APR-246 in controlling cell proliferation in most of the breast cancer cell lines tested, particularly the TNBC cell lines MDA-MB-231 and SUM159PT (Figure 2C). The TNBC cell line Hs578T, which exhibited less sensitivity toward CX-5461 (9.24 µM), also exhibited moderate sensitivity to this drug combination. When we increased the concentration of CX5461 to 2.5 µM, the cells showed much better sensitivity toward APR-246 (Figure S3A). Likewise, we found that increased concentration of CX-5461 enhanced the sensitivity of BT483 cells toward APR-246 (Figure S3B). The combination treatment did not affect the cell proliferation of near normal MCF10A or the luminal MCF7 with wild-type *TP53* (Figure S3C,D).

**Figure 3:**
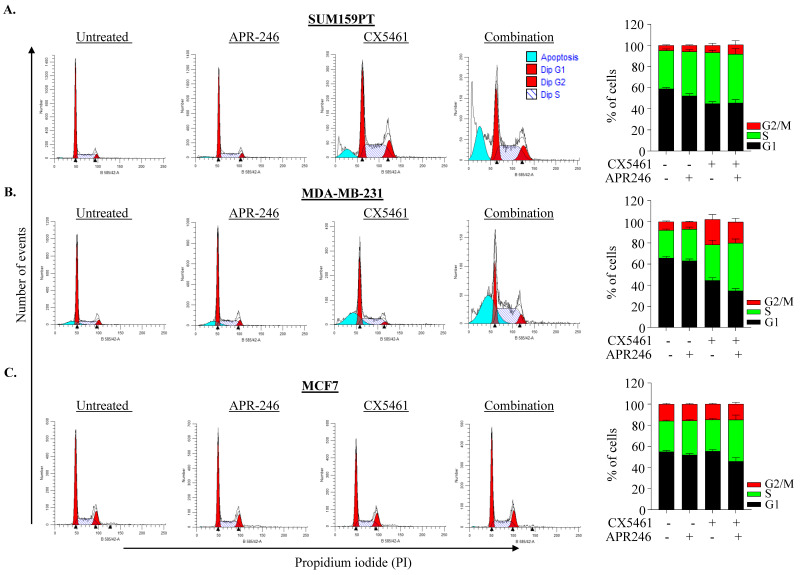
Impact of CX-5461 and APR-246 treatment on cell cycle perturbation. (**A**–**C**) Representative cytogram of SUM159PT (**A**), MDA-MB-231 (**B**) and MCF7 (**C**) cells showing cell cycle profiles following treatment with CX-5461 (0.5 μM) and APR-246 (25 μM) alone and in combination. Percentage of cell population in each phase of the cell cycle is shown. Graph represents the mean ± SD of two independent experiments.

We next assessed the effect of combination treatment on cell-cycle perturbation. Exposure of cells to 25 µM APR-246 for 48 h alone did not cause cell cycle perturbation when compared to untreated control, while a single 0.5 µM CX-5461 treatment marginally increased the cell percentage in S and G2/M-phases in SUM159PT and MDA-MB-231 cells. The combination treatment of APR-246/CX-5461 further increased S or G2/M phase cells in MDA-MB-231 cells but not in SUM159PT (Figure 3A–C). In MCF-7 cells, neither individual nor combination treatment resulted in significant cell-cycle perturbation (Figure 3C). Although combination treatment showed a reduction in cell proliferation; there was no consistent effect on cell-cycle perturbation upon single or combination treatment in these lines, suggesting that the effect on cell viability could be associated with cell death.

### 2.3. APR-246 and CX-5461 Combination Therapy Causes Apoptosis in a p21-Dependent Manner in TNBC Cells

**Figure 4:**
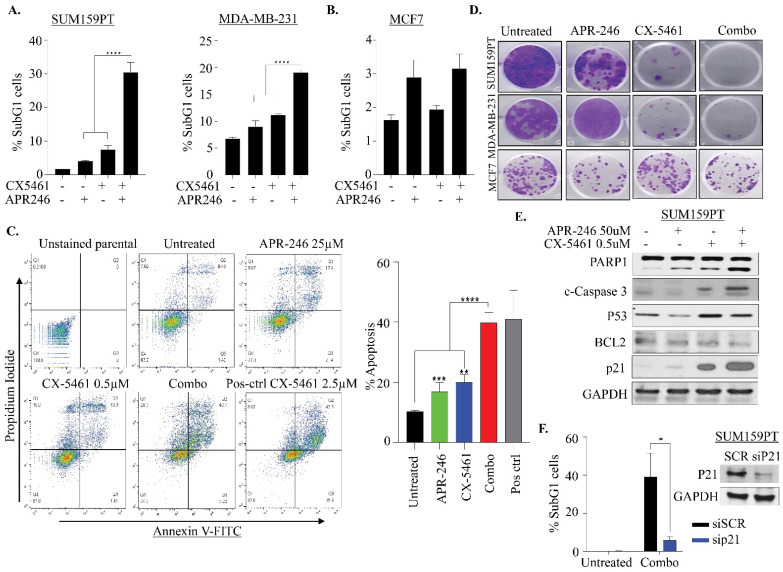
Impact of CX-5461 and APR-246 treatment on clonogenic survival and apoptosis. (**A,B**) Apoptotic fraction of sub-G1 population in SUM159PT, MDA-MB-231 and MCF7 determined by propidium iodide staining of cells treated with CX-5461 (0.5 μM) and APR-246 (25 μM) alone or in combination as described in of Figure 3. Graph represents the mean ± SD of two independent experiments. *** *p* ≤ 0.001. (**C**) Representative analysis images of SUM159PT cells treated with APR-246 (25 µM), CX-5461 (0.5 µM) alone and in combination for 48 h showing Annexin V/PI analysis. The top right quadrant represents the late apoptotic cells, while the bottom right quadrant the early apoptotic cells. Right: individual bar graph defines the additive number of cells in the late and early apoptotic stages. Cells treated with CX-5461 at 2.5 μM were used as positive control. Statistical analysis was carried out with one-way ANOVA and Dunnett’s multiple comparisons test relative to untreated parental. Mean ± S.D. *n* = 2. ** *p* ≤ 0.01; *** *p* ≤ 0.001; **** *p* ≤ 0.0001. (**D**) Representative images of colony-forming capacity of SUM159PT, MDA-MB-231 and MCF7 at 14 days in cells treated with APR-246 (50 µM) and CX-5461 (0.5 µM) alone and in combination, determined using crystal violet staining assays (*n* = 2). (**E**) Western blot analysis of expression of apoptotic pathway proteins. The SUM159PT cells treated with APR-246 (50 µM), CX-5461 (0.5 µM) alone and in combination for 24 h were analyzed for indicated protein expression. GAPDH was used as a loading control. (**F**) SUM159PT cells were reverse-transfected with 10 nM pooled p21 siRNAs for 48 h, followed by combination treatment with CX5461/APR-246 and apoptotic fraction of sub-G1 population was determined by propidium iodide staining, using flow cytometry. Mean ± S.D. *n* = 2. * *p* ≤ 0.05. Western blot image shows efficiency of P21 knockdown in SUM159PT cells.

In order to gain further into how combination therapy of APR-246 and CX-5461 might cause more pronounced growth suppression in TNBC cells, we analyzed the sub-G1 population of cells, which is indicative of the apoptotic population. Both SUM159PT and MDA-MB-231 cell lines showed an increase in the sub-G1 population upon combination treatment (Figure 3A,B and Figure 4A). SUM159PT cells were more sensitive to this combination treatment (~30% dead cells) compared to MDA-MB-231 (only 20% dead cells) (Figure 4A). However, we did not see any significant induction of death in MCF7 cells (Figure 3C and Figure 4B), which suggests that TNBC cells are sensitive to this drug combination, compared to luminal cells. We also performed Annexin V/PI staining in SUM159PT cells to determine if combination treatment drives cell death via apoptosis. Single treatment with either APR-246 (25 uM) or CX5461 (0.5 uM) for 48 h increased the apoptotic cell population by about ~9%, whereas combination treatment increased apoptosis by ~30% (calculated by subtracting apoptosis in untreated). Along with apoptosis, we noticed that cells treated with CX-5461 also exhibited an increase in necroptosis and which was further enhanced with combination treatment (Figure 4C). Thus, these data confirm that combination treatment triggers apoptosis and, to a lesser degree, necroptosis-mediated cell death in SUM159PT cells. To assess the anti-proliferative effects of combination therapy over a prolonged period, we next investigated the clonogenic potential of treated cells upon single and combined drug treatment. SUM159PT, MDA-MB-231 and MCF-7 were exposed to APR-246 (25 μM), CX-5461 (0.5 μM) and in combination for 24 h and reseeded in drug-free media for 14 days. Treatment of APR-246 alone had a lesser impact on the cell growth of SUM159PT and MDA-MB-231 in comparison to treatment with CX-5461 alone. Combined treatment led to a further decrease in the colony formation in these two lines (Figure 4D). However, in MCF-7, the number of colonies only slightly reduced in APR-246 monotherapy and in combination (Figure 4D).

To investigate whether the cell-cycle arrest and apoptosis were elicited by a p53-driven pathway upon APR-246 and CX-5461 combination therapy, proteins of the apoptotic and cell-cycle pathways were analyzed by immunoblotting. Both single agents induced cleaved PARP levels which substantially increased with combination treatment (Figure 4E). Likewise, we also found that cleaved Caspase 3 was more pronounced in combination treatment with reduction in BCL2 (Figure 4E), suggesting that the cell death is Caspase dependent. Protein p21 plays a pivotal role in cell-cycle arrest [34]. Strikingly, we found that p21 levels were markedly upregulated in both CX-5461 and combination-treated cells, while being only marginally so with APR-246 (Figure 4E). Therefore, we knocked down p21 using siRNA and found that a reduction in p21 levels abolished the apoptotic cell death seen with the combination treatment, assessed through subG1 analysis (Figure 4F). In addition, in order to investigate if the efficacy of combination therapy is solely observed in p53 mutant lines, we used p53 null MDA-MB-436 cells to assess the effect of combination therapy. Surprisingly, treatment of MDA-MB-436 cells with APR-246 alone caused massive cell death, assessed through subG1 and cleaved PARP (Figure S4A,C), thus nullifying the effect of CX-5461. We also noted that CX5461 caused S and G2/M arrest in these cells without apparent apoptosis. Likewise, we noticed no apparent changes in p21 levels with single and combination therapy, suggesting that APR-246 independently caused apoptosis by other means in MDA-MB-436 p53 null cells (Figure S4D). Collectively, these data suggest that APR-246 acts in both p53-mutant and null cell lines, and that CX5461 and APR-246 combination sensitivity is probably cell-line and context-dependent.

### 2.4. APR-246 and CX-5461 Combination Therapy Elicits DNA Damage and Replication Stress

**Figure 5:**
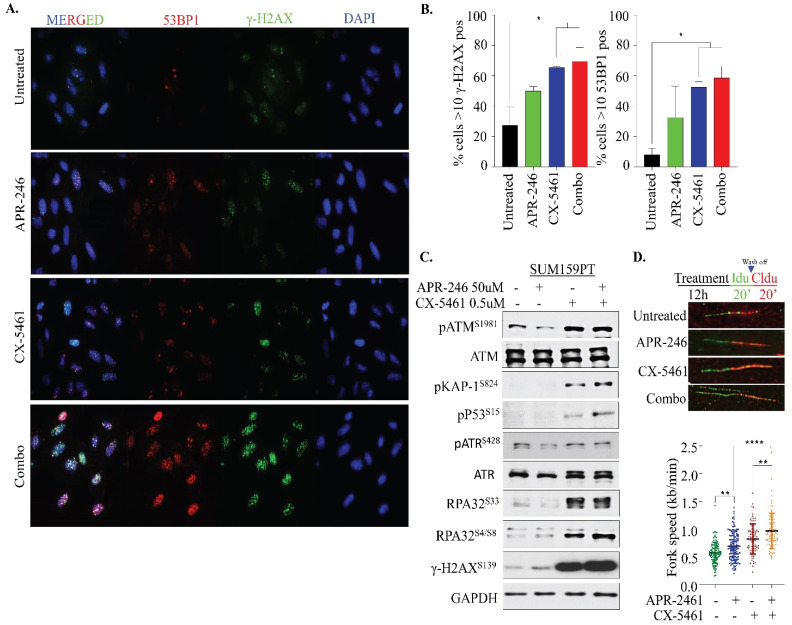
Impact of CX-5461 and APR-246 treatment on DNA damage and replication stress. (**A**) Immunofluorescence analysis of SUM159PT cells treated with APR-246 (25 µM) and CX-5461 (0.5 µM) as single agents and in combination for 12 h. The number of γ-H2AX (green) and 53BP1 (red) foci positive cells were determined. The augmented staining intensity can be correlated to the enhanced DNA damage response induced by the combination therapy. Fluorescence images are from representative fields for independent experiments (*n* = 2) carried out in duplicate. (**B**) Graph shows quantification of the percentage of cells with > 10 γH2AX and 53BP1 foci. Statistical analysis was performed using one-way ANOVA and Dunnett’s multiple comparisons test. * *p* ≤ 0.05 (**C**) Western blot analysis of expression of DNA damage response signaling proteins. The SUM159PT cells treated with APR-246 (50 µM), CX-5461 (0.5 µM) alone and in combination for 24 h were analyzed for indicated protein expression. GAPDH was used as a loading control. (**D**) Schematic and representative images of DNA fiber, and quantification of the fork seeds (kb/min) in SUM159PT cells treated APR-246 (50 µM), CX-5461 (0.5 µM) alone and in combination for 12 h. ** *p* ≤ 0.01; **** *p* ≤ 0.0001.

A previous report with CX-5461 as a single agent showed the drug’s efficacy in activating the ATM cascade and a G2/M arrest [35], while APR-246 activates oxidative stress-response signaling by inducing ROS [22,23,24]. Increased intracellular oxidative stress is known to induce DNA damage in cells. Therefore, we investigated DNA damage response (DDR) signaling upon combination treatment in our model. Many repair proteins localize to discrete hubs of DNA repair in response to damaging agents called damage-induced foci, which can be readily visualized by immunofluorescence (IF). As the formation of foci directly correlates with the amount of DNA damage and repair, it provides a reliable quantitative measure of the dynamics of DNA repair. To evaluate the effects of single and combination drug treatments in this experimental setting, γ-H2AX and 53BP1 foci were analyzed in SUM159PT cells. Treatment of cells with both APR-246 and CX-5461 for 12 h caused accumulation of both 53BP1 and γ-H2AX foci, albeit with much higher immunoreactivity intensity upon combination treatment (Figure 5A,B), suggesting that combination therapy was effective in eliciting DNA damage in our model. To further illustrate DDR pathway activation at a molecular level, SUM159PT cells were treated with APR-246 and CX-5461 in monotherapy and in combination for 24 h, followed by the analysis of various DNA damage proteins by immunoblotting. Notably, a massive induction of γ-H2AX levels was detected in the SUM159PT cells treated with CX-5461 as a monotherapy, which was further increased upon combination with APR-246 but not with the APR-246 individual treatment (Figure 5C). ATM is known to be recruited to sites of DSBs. Accordingly, we found activation of ATM signals indicated by phosphorylation at the S1981 site in SUM159PT upon combination treatment and CX-5461 monotherapy, while APR-246 alone did not cause a significant increase in phosphorylated ATM. Another marker of the DDR response is phosphorylated KAP1, a direct target of ATM upon DNA damage, which was analyzed in response to drug treatments. The upregulation of phosphorylated KAP1 expression was observed in SUM159PT treated with CX-5461 individually and in the combination therapy (Figure 5C). However, no significant increase in pKAP1 was seen in the individual APR-246 treatment, suggesting that DNA damage is more prevalent with CX-5461 than APR-246. Similarly, replication stress markers’, RPA32 S33 and S4/S8, phosphorylation levels were strongly upregulated for both the combination treatment as well as the CX-5461 individual treatment, but not with APR-246 monotherapy. To corroborate increase in RPA32 phosphorylation to replication stress-mediated cell killing upon combination treatment, we performed a DNA fiber assays to determine the progression of replication forks (Figure 5D). First, we treated the cells with single and combination therapy for 12 h, followed by labeling with thymidine analogs (either 5-Iodo-2′-deoxyuridine (IdU) or 5-chloro-2′-deoxyuridine (CldU)) as shown in Figure 5D. Surprisingly, we found that both drug treatments caused a significant increase in replication fork speeds individually which was further enhanced in combination. Taken together, this observation suggests that DNA damage elicited by combination therapy by APR-246 and CX-5461 causes hyper-replicating forks, which may account for cell death in our model.

## 3. Discussion

This study provides evidence for the upregulation of RNA Pol I in breast cancer, particularly basal-like breast cancer, which is also associated with poor clinical outcomes as well as a possible synergistic interaction between targeting RNA pol I machinery using CX-5461 with p53 activator APR-246 in TNBC cell lines. CX-5461 is currently in clinical trials for BRCA mutant breast cancer in Canada (Clinical trial identification: NCT02719977), while in hematological cancers, it has shown antitumor activity with a good safety profile [36]. Very recently however, several studies have showed that CX-5461 can act as a DNA G-quadruplex stabilizer as well as topoisomerase II poison, arguing an effect of CX-5461 beyond Pol I inhibition as previously anticipated [30,33]. APR-246, which is clinically effective in prostate and hematological cancers [37], was also shown to inhibit growth and metastatic potential of TNBCs by several studies [38,39]. Although APR-246 has been well studied in the context of mutant p53, more recent data suggests that it has an independent role in both wild-type and p53 null contexts [22,40]. Notably, both inhibitors as single agents, under our study conditions, failed to show either a mutant P53- or subtype-specific association. Interestingly however, TNBC cell lines were substantially more sensitive to the combination treatment in our study, suggesting that the synergistic effects observed between these two drugs can possibly be exploited to target TNBC in particular. Consistent with this idea, multiple studies have shown the synergistic effects of combining APR-246 or CX-5461 with DNA-damaging drugs as a potential treatment strategy for multiple cancer treatments [24,41,42,43,44,45].

In this study, we showed that a CX-5461 and APR-246 drug combination significantly suppressed cell growth and induced cell death in TNBC cell lines. The growth suppression and cell death observed in these cells seems to be driven by multiple mechanisms. In particular, we found an increase in cell death upon combination treatment through Caspase-dependent apoptosis. In line with our observation, Caspase-driven apoptosis mediated by APR-246 was shown in breast and lung cancer cells [39,46] along with autophagy in sarcoma, breast and colon cancer cells [47,48]. Likewise, studies with osteosarcoma, melanoma and pancreatic cells have also showed that CX-5461 drives cell death mainly through autophagy [28,49] and, to a lesser extent, through Caspase-dependent apoptosis in acute lymphoblastic leukemia cells [50]. However, we did not evaluate autophagy in our setting. 

Mechanistically, we found that both drugs elicit DNA double-strand breaks, as evaluated by gamma H2AX, but only CX-5461 evoked ATM-driven DNA damage signaling within the cells. Previous studies have shown that treatment with CX-5461 causes substantial cellular DNA damage, leading to activation of the ATM/ATR signaling pathway, which, in turn, activates downstream CHK2/CHK1, resulting in G2/M arrest [50,51,52]. However, in our study conditions, the G2/M arrest was not so apparent between the cell lines tested. Notably, a greater increase in S4/S8 phosphorylation of RPA32, which protects single-strand DNA (ssDNA), as well as S33 phosphorylation of RPA32 suggest that the CX-5461 treatment results in replication stress, leading to stalled forks as previously shown by others [53]. Accumulation of an S phase cell fraction in SUM159PT following treatment also proves ongoing replication stress. Interestingly, we observed that treatment with both APR-246 and CX-5461 led to acceleration of the fork speed in SUM159PT cells, which is further exacerbated upon combination treatment. Accelerated fork elongation is proven to be a mechanism for replication stress and DNA damage as demonstrated by several recent studies on PARP inhibitors [54,55]. Thus, our data demonstrate that both APR-246 and CX-5461 cause replication stress, possibly via similar effects but to varying extents, on fork progression. In contrast, in a recent ovarian cancer study [54], CX-5461 seemed to have no effect on fork speed at 0.1 µM compared to 0.5 µM used in our study, suggesting the drug concentration could play a major role in replication stress. Overall, our study has shown that the growth inhibition observed in TNBC cells following the combination treatment is result of the cumulative effects of DNA damage, replication stress and cell death.

Despite intensive research and the discovery of therapeutic drugs targeting TNBC, the potential of the subtype specific-targeted therapy remains largely unsuccessful. The development of resistant tumors upon chemotherapy and target rewiring in cancer cells are just a few of the many limitations. Mutation in *TP53* in the majority of TNBC cells is a major determinant of resistance to many chemo- and targeted therapies. Moreover, TNBC cells are very much heterogeneous in nature and give rise to resistant clones that hinder the effectiveness of these therapies. Given the varying responses to mono and combination therapy in this study, it is imperative that this study be further extended to a large panel of breast cancer cell lines and in vivo models. This will not only assist in the identification of underlying molecular mechanisms and patterns, but also determine the therapeutic significance of this drug combination in treating TNBC.

## 4. Materials and Methods

### 4.1. Reagents and Antibodies

CX-5461 and APR-246 were purchased from Selleck Chemicals LCC. Both drugs were prepared in organic solvent, DMSO, and stored at −80 °C. Small interfering RNAs (siRNAs) were obtained from Shanghai GenePharma (Shanghai, China). Lipofectamine RNAiMAX was purchased from Life Technologies (Waltham, MA, USA). The CellTiter 96^®^ AQueous One Solution Cell Proliferation Assay kit was purchased from Promega Corporation (Madison, WI, USA). The antibodies used in this study are listed in Table S1.

### 4.2. Cell Culture

The breast cancer cell lines were obtained from the American Type Culture Collection (ATCC, Manassas, VA, USA). Cells were cultured in DMEM (Dulbecco’s Modified Eagle’s Medium–4.5 g/L glucose, (Sigma Aldrich^®^, Saint Louis, MO, USA) with 10% fetal bovine serum (FBS) as described previously [56,57,58].

### 4.3. Cell Viability Assay

The cell viability assays were performed using the CellTiter^®^ 96 aqueous cell proliferation assay reagent (Promega Corporation, Madison, WI, USA) as described previously [56,57,58]. The experiment was performed in triplicate for all treatment conditions. Cells were plated at a density of 1000 cells per well with a total volume of 200 µL in a 96-well tissue culture plate. Drug treatment was performed after 24 h and the cells were incubated for a period of 6 days. On the sixth day, 100 µL of media containing 10% MTS reagent was added to each well under low-light conditions and the plate was incubated for 1 h, prior to reading at 490 nm with a Biorad Benchmark Plus microplate spectrophotometer.

### 4.4. Immunoblotting

For Western blotting, cells were seeded onto 100 mm sterile tissue culture dishes at a seeding density of 1 × 10^6^/dish, with a final volume of 6 mL per dish and incubated overnight. The following day, the cells were administered the drug treatment and incubated for a further period of 24 h. Subsequently, the cells were harvested by scraping in ice cold 1X PBS and centrifuged at 2400 rpm for 5 min at 4 °C. The lysates were resuspended in urea buffer and sonicated for 10–15 s. Protein quantification was determined using the Bicinchoninic acid (BCA) assay according to the manufacturer’s instructions (Pierce^TM^ BCA Protein Assay Reagent A&B), with BSA as the standard. For preparing Western blot protein samples, 30 µg of lysate was combined with 5X Laemmli protein loading dye prior to loading. The samples, plus a protein ladder of known molecular weight (PageRuler Prestained, ThermoScientific^®^,Waltham, MA, USA), were electrophoresed at 80 V for 10 min and subsequently at 120 V in a 1X Tris-SDS-glycine running buffer. The proteins were transferred onto an Amersham Hybond C nitrocellulose membrane (GE Healthcare, Chicago, IL, USA), at 100 V for 90 min in an ice-cold 1X transfer buffer, blocked in 5% skim milk for 1 h at room temperature, and incubated overnight with primary antibodies at 4 °C. The next day, the membranes were rinsed with PBST three times, incubated with secondary antibodies for 1 h at room temperature and rinsed further with PBST. The protein bands were detected, using enhanced chemiluminescence (ECL) plus (PerkinElmer) on X-ray films with LAS-4000 imaging system (Fujifilm Life Sciences, Cambridge, MA, USA).

### 4.5. Cell Cycle Analysis

For the cell cycle analysis, 1 × 10^5^ cells were seeded in 6-well tissue culture plates and treated with drugs the following day. After 72 h the supernatant, the cells were collected by trypisinization, centrifuged and pelleted. The pellet was resuspended and washed twice, using freshly prepared, ice-cold 1 mL of 1% FBS in PBS and centrifuged again at 2400 rpm for 5 min at 4 °C. The cells were fixed with 4 mL of ice-cold 100% ethanol while vortexing. The ethanol was completely removed by centrifuging the cells at 2400 rpm for 5 min at 4 °C. The samples were resuspended in 5 mL of 1% FBS in PBS and washed at 2400 rpm for 5 min at 4 °C. Lastly, the cells were stained by adding 360 µL of staining solution (150 µL of 1 mg/mL propidium-iodide (PI; Sigma Aldrich^®^ Saint Loius, MO, USA) plus 37.5 µL of 10 mg/mL RNAaseA in 15 mL of 1%FBS/PBS). The samples were incubated for 30 min at 37 °C and protected from light. The fluorescence spectra were detected by Fluorescence Activated Cell Sorting CANTO II at 585 nm with BD FACSDIVA v 8.0 software (BD Biosciences). Further analysis of the raw data was performed, using ModFit LT v 4.0 (Verity Software, Bengaluru, Karnataka, India) and GraphPad Prism v 7.0a (San Diego, CA, USA) for the cell-cycle profile.

### 4.6. Colony Formation Assay

Cells treated with drugs for 24 h were seeded at a density of 1000 cells per well of a 12-well tissue culture plate. The expended media were discarded and replaced with fresh media. For fixation and staining, 1 mL of 0.05% crystal violet stain containing methanol was added to each well and kept on a shaker for 30 min at room temperature. The stain was discarded, and the plate was washed under running water.

### 4.7. Immunofluorescence

For the immunofluorescence assays, SUM159PT cells were seeded at a density of 1 × 10^5^ in a 6-well tissue culture plate containing sterile and UV-irradiated cover slips. The treated cells were harvested after 12 h and washed once with 1X PBS. The adhered cells were stripped of the cytoplasm with CSK buffer exactly for 1 min and subsequently fixed in ice-cold 4% paraformaldehyde (PFA) (Sigma Aldrich^®^) in PBS for 45 min. Afterward, the cells were permeabilized with 0.5% TritonX-100 (Sigma Aldrich^®^) in PBS for 45 min at room temperature and blocked with filtered 3% BSA (Sigma Aldrich^®^) in PBS for another 45 min at room temperature. Coverslips from individual treated conditions were incubated with a primary antibody cocktail in a humidified chamber at 37 °C for 1 h. Next, the cells were washed thrice with 0.5% TritonX-100 and incubated with a secondary antibody cocktail and DAPI nuclear stain (1:300) in a humidified chamber at 37 °C for 1 h in the dark. Following secondary antibody incubation, the coverslips were washed with TritonX-100 thrice. These coverslips were mounted on slides, using the Prolong™ Gold antifade mounting medium (ThermoScientific^®^,Waltham, MA, USA). The analysis of immunofluroscence in drug-treated cells was performed using Delta Vision personal DV deconvolution microscope (Applied Precision, GE Healthcare, Issaquah, WA, USA). The images were analyzed using Fiji ImageJ software v. (Java3D, Austin, TX, USA).

### 4.8. AnnexinV/PI Staining

For determining apoptosis, the cells were plated at a density of 1 × 10^5^ in duplicate in 6-well tissue culture plates. The next day cells were treated with drugs and incubated for 48 h. The cells were trypsinized, centrifuged at 2400 rpm for 5 min at 4 °C and washed once in 1X PBS. The pellet was gently resuspended in 100 µL of 1X binding buffer, diluted from 10X binding buffer (0.1 M HEPES/NaOH (pH 7.4), 1.4 M NaCl, 25 mM CaCl2) and 5 µL of Annexin V-FITC conjugate. The cells were incubated for 15–20 min on ice and protected from light. Next, 5 µL of PI (1 mg/mL) and 400 µL of binding buffer were added to each sample. The parental samples were left unstained. Fluorescence was detected instantly on a BD FACS Canto II at 530 and 670 nm, respectively, using BD FACS DIVA v. 8.0. The analysis was performed using FlowJo v. 10.0.6 (Tree Star, Ashland, Oregon, USA).

### 4.9. DNA Fiber Assay

The DNA fiber protocol was performed as previously described by us and others [58,59,60]. The cells were labeled with CldU and IdU for 20 min each. The progressive replication fork speed was calculated based on the length of the CldU tracks measured, using ImageJ software. At least 100 replication tracks were analyzed for each sample. The fork speed was calculated based on a conversion factor of 1 µm = 2.59 kb [60].

### 4.10. Statistical Analysis

One-way or two-way ANOVA with Tukey or Bonferroni’s testing was performed, using GRAPHPAD PRISM v 7.0a (GraphPad Software, La Jolla, CA, USA) and the *p*-values were calculated as indicated in the corresponding figure legends. Asterisks indicate significant difference and are represented as: * *p* < 0.05, ** *p* < 0.01, *** *p* < 0.001 and **** *p* < 0.0001, ns = not significant.

## Data Availability

The datasets generated and/or analyzed during the current study are included in this published article (and its supplementary information files) and all the raw data are available from the corresponding author upon reasonable request.

## Supplementary Materials

The following are available online at https://www.mdpi.com/article/10.3390/ijms22115782/s1.
