# Supplementary material for: CX-5461 Enhances the Efficacy of APR-246 via Induction of DNA Damage and Replication Stress in Triple-Negative Breast Cancer
Source: Int J Mol Sci. 2021 May 28;22(11):5782. doi: 10.3390/ijms22115782 (PMC8198831; doi:10.3390/ijms22115782)
Supplement: Supplementary file 1 [file ijms-22-05782-s001.zip › ijms-1239191-supplementary.pdf]

International Journal of *Molecular Sciences*

MDPI

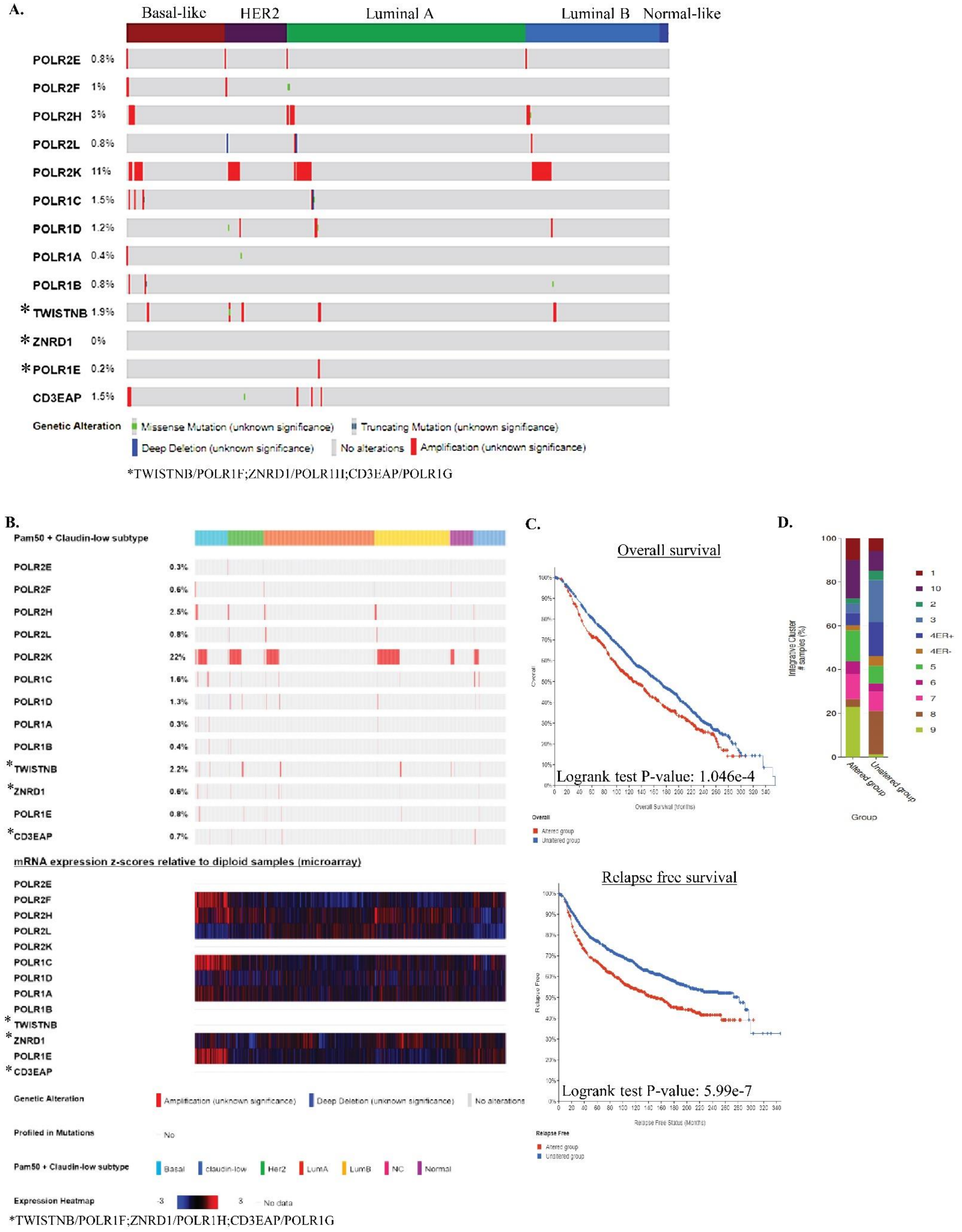

Figure S1: RNA polymerase I transcription alteration in breast cancer. Percentage indicative of multiple alteration in components of RNA Pol 1 transcription in TCGA breast cancer samples. **(A)** and METABRIC breast cancer samples **(B). (B, lower panel)** Heatmap analysis of components of RNA Pol 1 transcription at mRNA levels in METABRIC breast cancer samples. Patient samples were divided into PAM50+Claudin-low subtype. (**C)** Breast cancer METABRIC patient's data was divided into two subgroups based on their expression patterns (whether altered or non-altered) and survival probability (overall and relapse free survival) was plotted using Kaplan-Meier survival analysis.
(D) Integrative cluster analysis of patients with and without Pol I transcription alteration determined using METABRIC dataset.

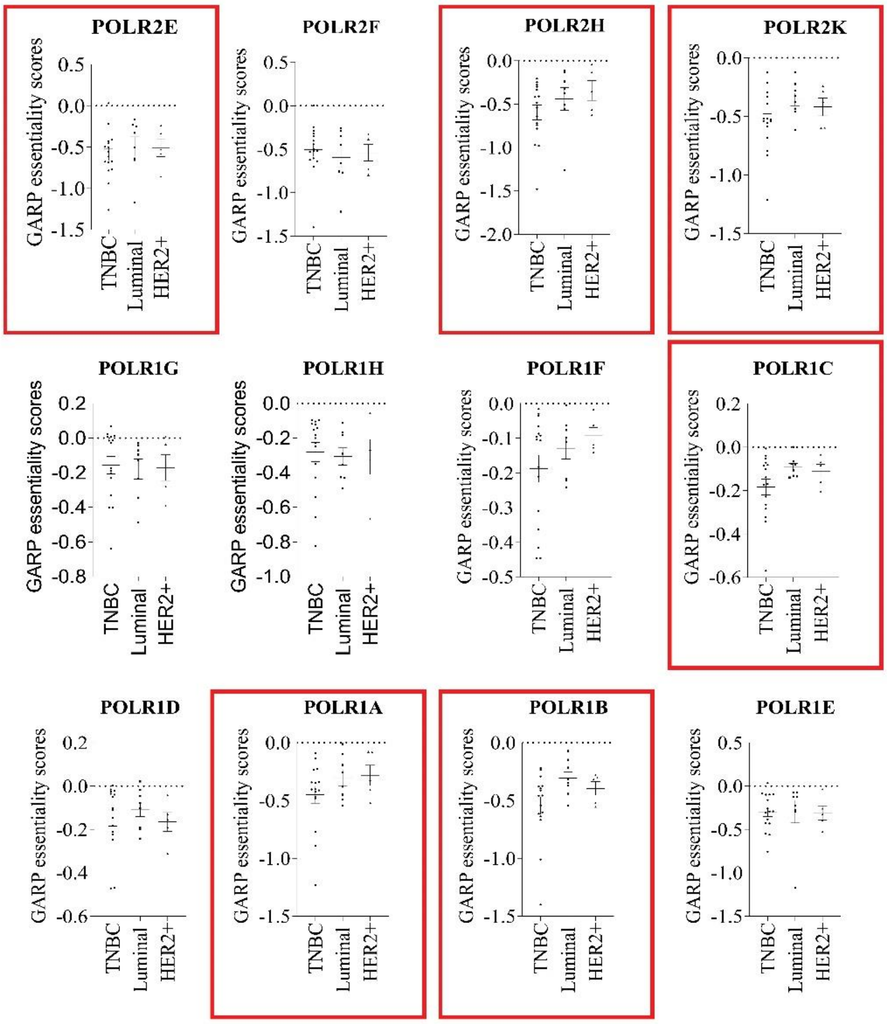

Figure S2: Impact of shRNA knockdown of RNA Pol I components in breast cancer cell lines. Normalised zGARP essentiality score for RNA Pol I genes displaying differential essentiality in specific breast cancer subtypes. The data derived from study from Marcotte et al.[1]

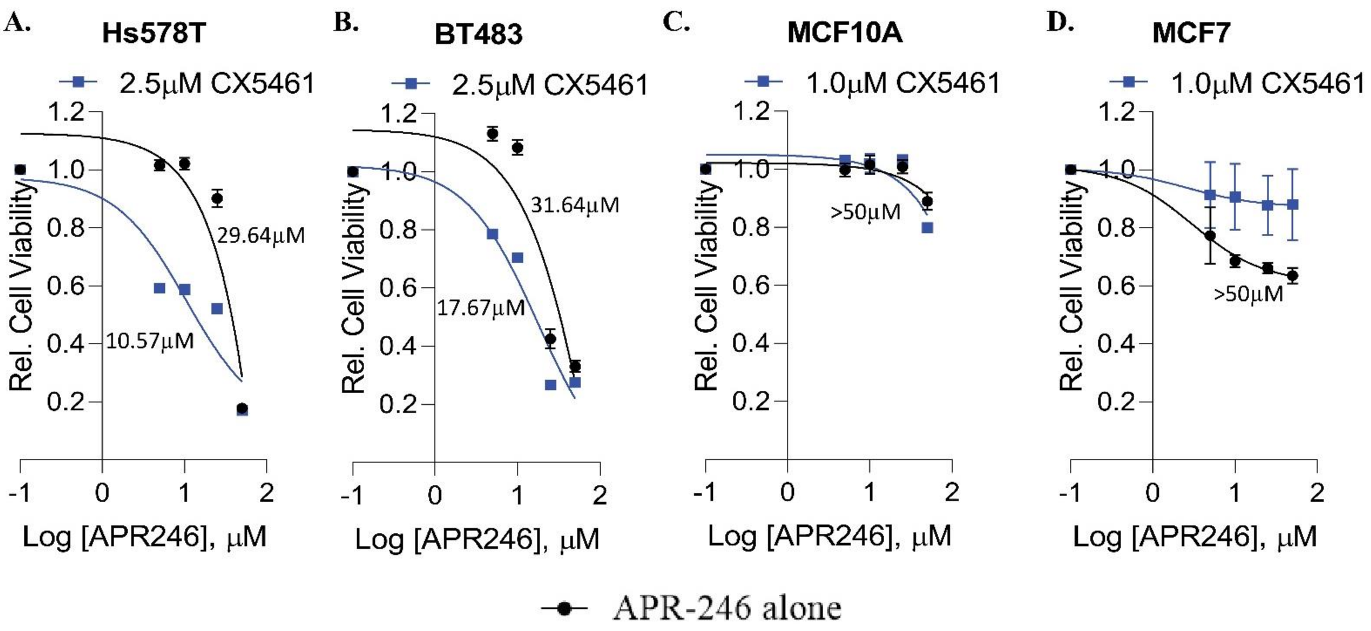

Figure S3: Impact of CX5461 and APR-246 treatment on cell growth in a panel of breast cancer. **(A-D)** Representative cell lines as indicated in the figure were exposed to different concentration of APR-246 (5-25 μM) alone or in combination with 1 or 2.5 μM of CX-5461 and cell viability was determined as described in Figure 2.

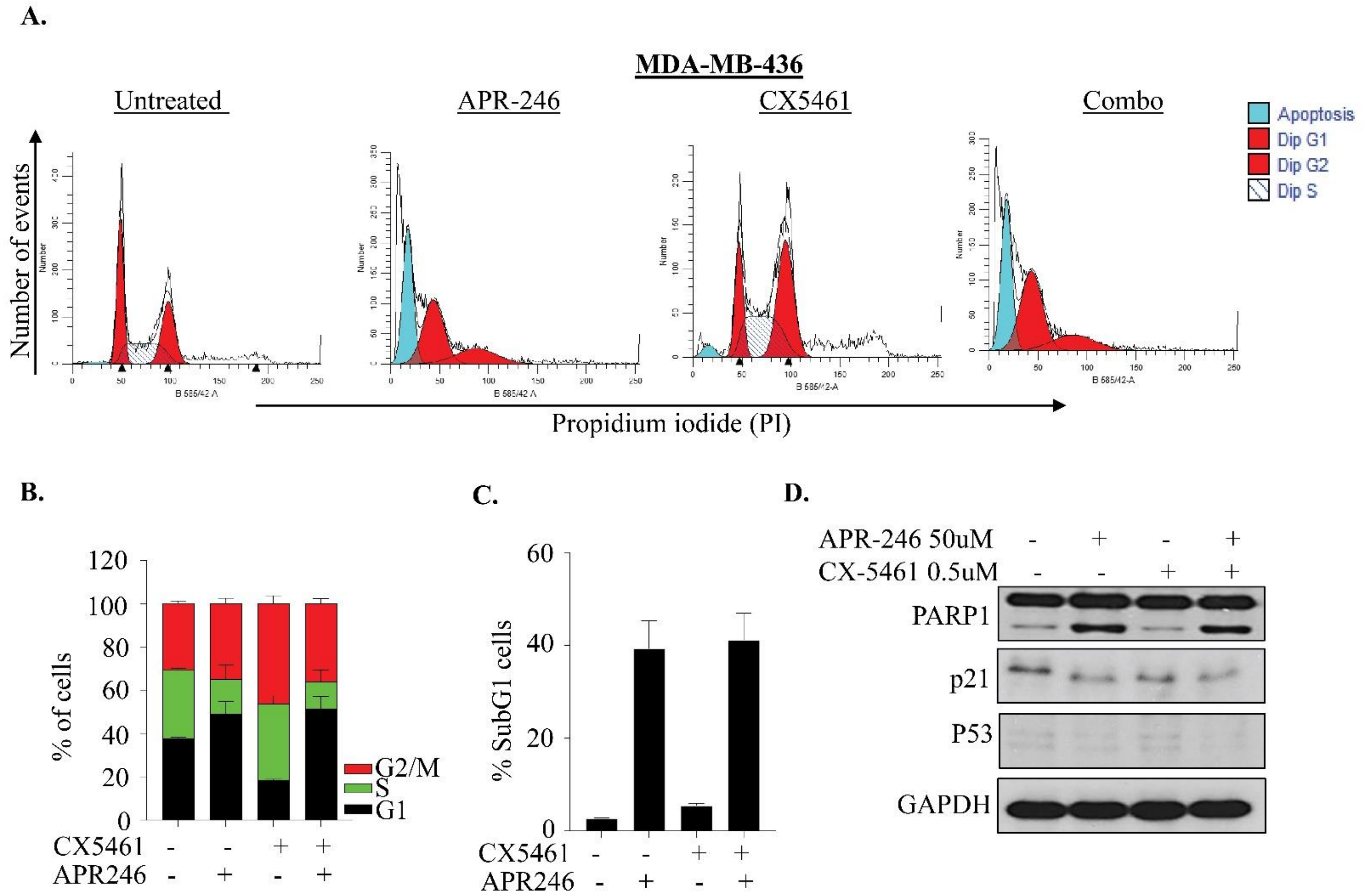

Figure S4: Impact of CX5461 and APR-246 treatment on cell growth, cell cycle perturbation and apoptosis in MDA-MB-436. (A) Representative cytogram of control and treated MDA-MB-436 cells showing cell cycle profiles following treatment with CX-5461 (0.5 µM) and APR-246 (25 µM) alone and in combination. Percentage of cell population in each phase of cell cycle is shown in **B.** Graph represents the mean ± SD of two independent experiments. **(C)** Apoptotic fraction of sub-G1 population determined by propidium iodide staining as described in panel figure 4A. **(D)** Western blot analysis of expression of apoptotic pathway proteins following APR-246 (50µM), CX-5461 (0.5µM) treatment for 24 h. GAPDH was used as a loading control.

Table S1: List of antibodies used in this study.

**Antibodies for Western blotting**				
Antibody	**Species**	**Manufacturer**	**Cat. No.**	**Dilution**
Anti-CHK2 clone 7	Mouse	EMD Millipore	2763965	1:1000
ATM Total	Mouse	Abcam	GR259127-5	1:1000
ATR Total	Goat	Santa Cruz Biotechnology	E1012	1:500
Bcl-2	Mouse	Cell Signaling Technology	3	1:1000
GAPDH	Rabbit	Trevigen	2275-PC-100	1:1000
p p53 S15	Rabbit	Cell Signaling Technology	19	1:1000
p21	Mouse	BD Biosciences	556431	
p53 Total	Mouse	Santa Cruz Biotechnology	K1914	1:250
PARP-1	Rabbit	Cell Signaling Technology	15	1:1000
pATM S1981	Rabbit	GeneTex	42340	1:1000
pATR S428	Rabbit	Cell Signaling Technology	3	1:1000
pKAP-1 S824	Rabbit	Bethyl	A300-767A-T	1:1000
γH2AX	Mouse	EMD Millipore	2943815	1:1000
RPA 32 S33	Rabbit	Bethyl	A300-246A	1:1000
RPA 32 S4/S8	Rabbit	Bethyl	A300-245A	1:1000
**Antibodies for Immunofluorescence**				
Antibody	**Species**	**Manufacturer**	**Cat. No./ Lot No.**	**Dilution**
γH2AX	Mouse	EMD Millipore	2943815	1:1000
53BP1	Rabbit	Novus Bio	D-12	1:1000
Alexa Fluor™ 488-donkey	Mouse	Invitrogen Thermofisher Scientific®	A21202	1:300
Alexa Fluor™ 546-goat	Rabbit	Invitrogen Thermofisher Scientific®	A11010	1:300

Figure 4E

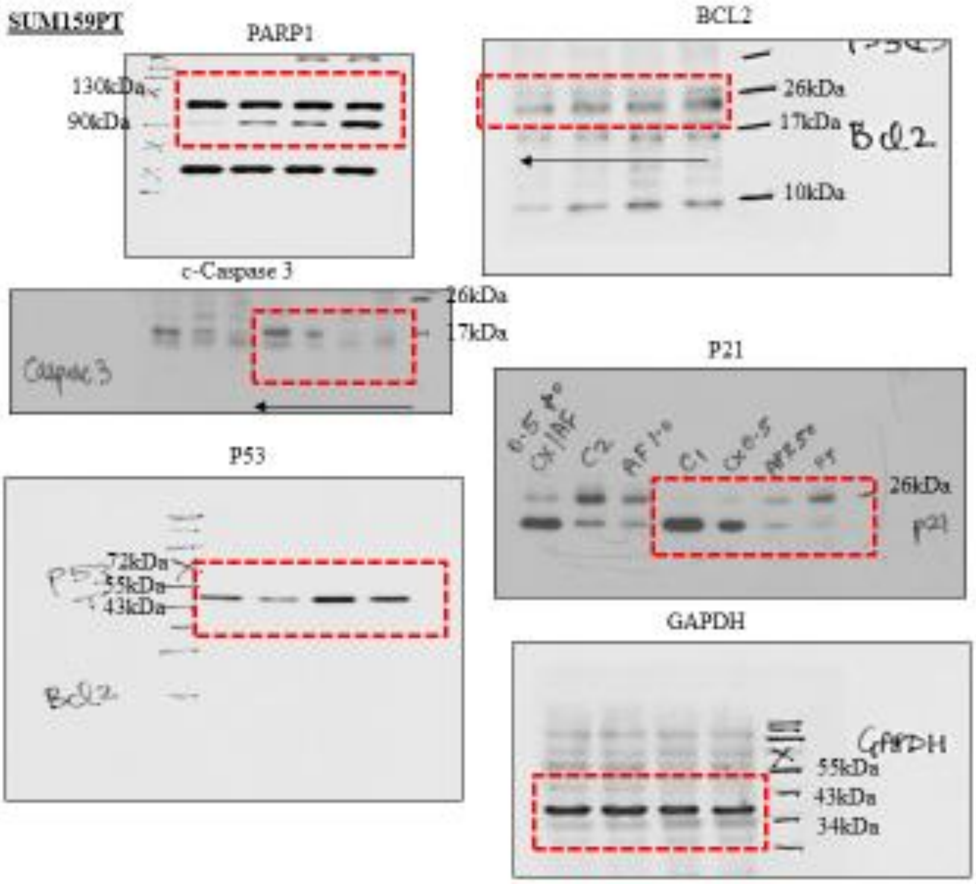

Figure 4F

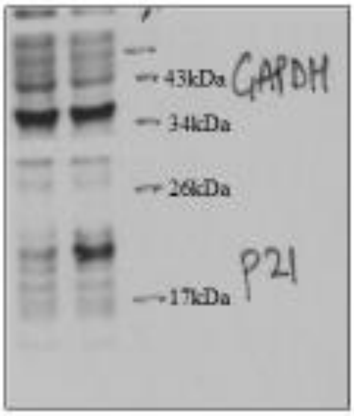

Uncropped images for Figure 4E and 4F

Figure S4D

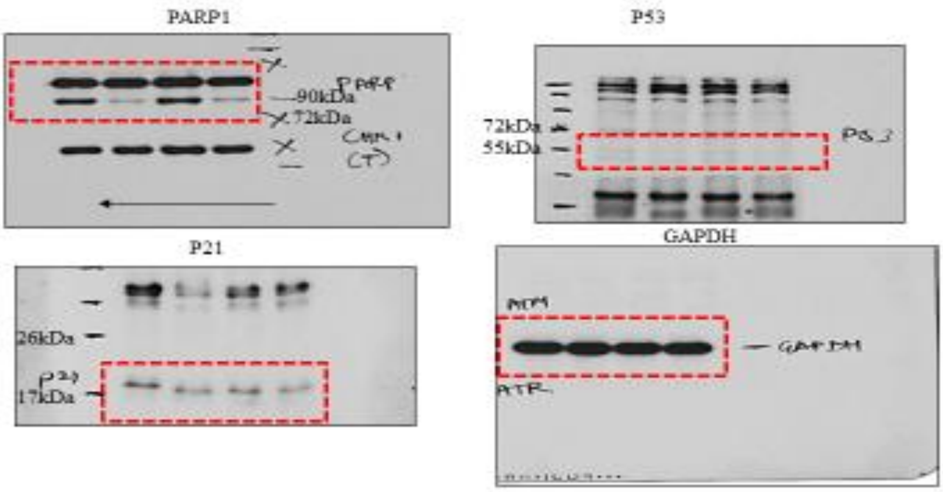

Uncropped images for Figure S4D

Figure 5C

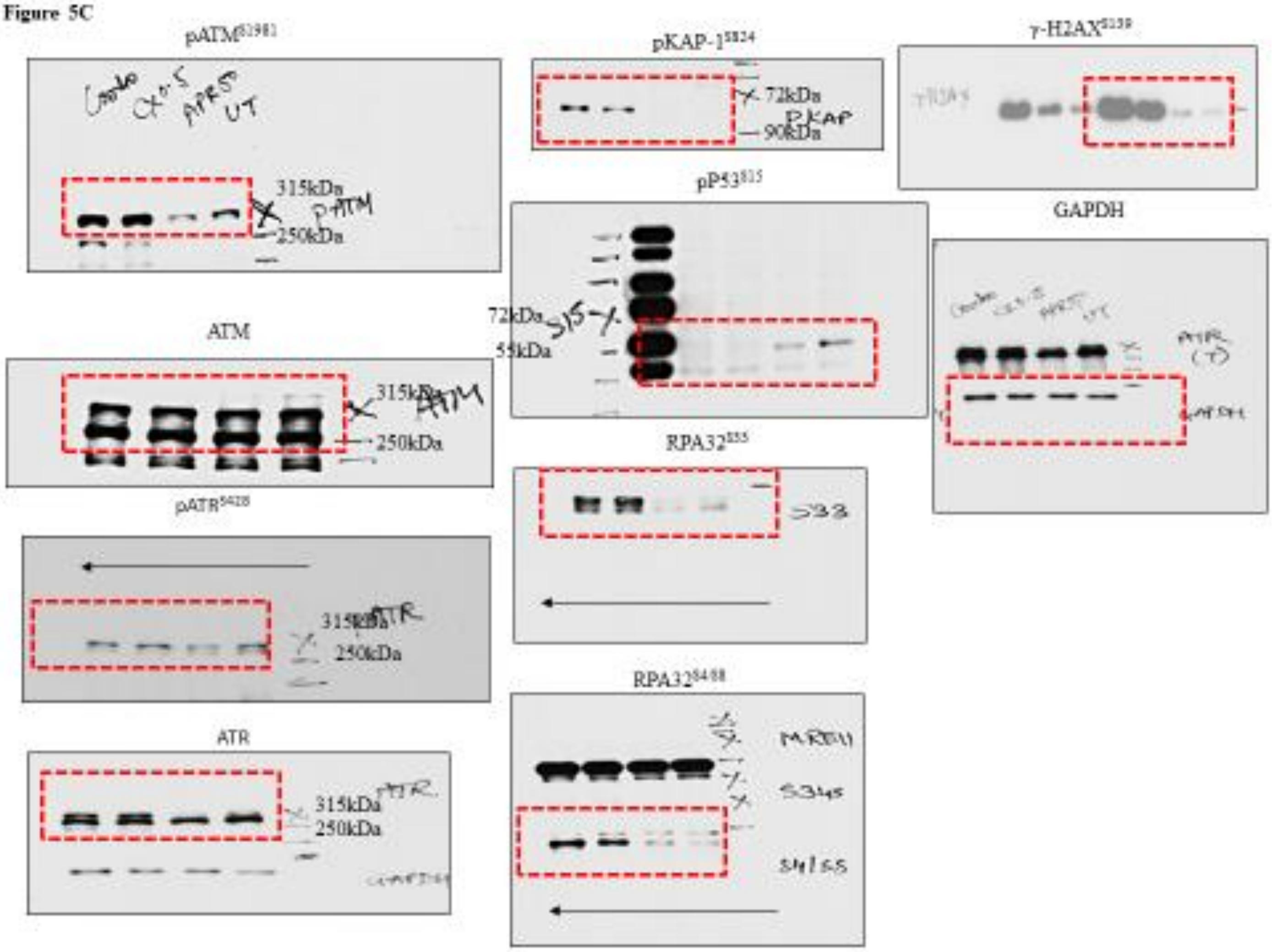

Uncropped images for Figure 5C

References.

1. Marcotte, R.; Brown, K. R.; Suarez, F.; Sayad, A.; Karamboulas, K.; Krzyzanowski, P. M.; Sircoulomb, F.; Medrano, M.; Fedyshyn, Y.; Koh, J. L. Y.; van Dyk, D.; Fedyshyn, B.; Luhova, M.; Brito, G. C.; Vizeacoumar, F. J.; Vizeacoumar, F. S.; Datti, A.; Kasimer, D.; Buzina, A.; Mero, P.; Misquitta, C.; Normand, J.; Haider, M.; Ketela, T.; Wrana, J. L.; Rottapel, R.; Neel, B. G.; Moffat, J., Essential gene profiles in breast, pancreatic, and ovarian cancer cells. *Cancer Discov* **2012,** 2, (2), 172-189.
